# Correlation Between Renal Calculus Disease and Iliac Bone Thickness on a Single Non-contrast CT Scan

**DOI:** 10.7759/cureus.40965

**Published:** 2023-06-26

**Authors:** Padma V Badhe, Dinesh Shinde, Sambhaji Pawal, Ajith R Varrior, Moinuddin Sultan, Gautham Shankar

**Affiliations:** 1 Department of Radiology, Seth Gordhandas Sunderdas Medical College and King Edward Memorial Hospital, Mumbai, IND; 2 Department of Radiodiagnosis, Seth Gordhandas Sunderdas Medical College and King Edward Memorial Hospital, Mumbai, IND; 3 Department of Interventional Radiology, Dr. D. Y. Patil Medical College and Research Centre, Pune, IND; 4 Department of Radiology, Vedantaa Institute of Medical Sciences, Dahanu, IND

**Keywords:** calcium, bone density, computed tomography, iliac bone thickness, renal calculus

## Abstract

Background

Renal calculi remain a major economic and health burden worldwide and are considered a systemic disorder associated with multiple other diseases. Several studies have observed that patients with idiopathic calcium renal stones show a reduction in bone mass. This study aimed to evaluate bone mass reduction on a non-contrast CT scan study in a group of idiopathic calcium-containing renal calculus disease patients in comparison with subjects without renal calculus disease.

Methodology

This non-interventional, cross-sectional study included a total of 300 patients with 150 cases (with renal calculi) and 150 controls (without renal calculi). Patients were divided according to age groups of 18-40, 40-60, and more than 60 years. The renal calculus size and the mean iliac bone thickness were calculated, and Spearman’s correlation test was used to determine the correlation between them.

Results

The mean iliac bone thickness was significantly lower in the cases (3.29 mm) compared to the controls (9.73 mm with a standard deviation of 1.341 mm). There was a statistically significant negative correlation between the size of the renal calculus and the mean iliac bone thickness.

Conclusions

Renal calculus disease associated with hypercalciuria caused by increased bone resorption is reflected by the decreased iliac bone thickness on CT scans. Our study used the iliac bone (cancellous bone) in predicting bone mass reduction which shows changes early in the course of the disease compared to the neck of the femur and lumbar vertebrae (compact bones). It helps in predicting osteoporosis early and prevents the progression of the disease through early and appropriate clinical and urological intervention.

## Introduction

Renal calculi remain a major economic and health burden worldwide. Renal calculus is a systemic disorder associated with multiple other conditions, including those of renal and other cardiac and skeletal diseases. There is an increased risk of coronary artery disease, hypertension, and metabolic syndrome. It typically presents in the middle age group and is more prevalent in the warmer climate due to more concentrated urine. Calcium is the major component (80%) in renal calculi, either as calcium oxalate (70%) or calcium phosphate (10%). High urinary calcium is the single most common abnormality of urine biochemistry in recurrent calculi disease [1].

It has been disputed whether hypercalciuria originates from increased calcium mobilization from bone or reflects increased intestinal calcium absorption [2]. In younger populations, kidney stones are more prevalent in those with primary hyperparathyroidism due to enhanced synthesis of 1,25 di-hydroxy vitamin D with intact kidney function, and consequent increased intestinal calcium absorption [3]. Bone disease may occur in older individuals due to their lower serum 1,25 di-hydroxy vitamin D levels and diminished intestinal calcium absorption.

Renal leak hypercalciuria is a second, less common variety of hypercalciuria in which defective renal tubular calcium reabsorption is accompanied by enhanced parathyroid hormone (PTH), calcitriol, and net intestinal calcium absorption [4]. Several studies have observed that idiopathic calcium renal stone formers show a bone mass reduction [5-8]. Moreover, the Jaegers group found that bone mass was reduced in all patients with renal calculus disease, independent of hypercalciuria [9-11].

The aim of this study is the evaluation of bone mass reduction on a non-contrast CT scan study in a group of idiopathic calcium-containing renal calculus disease patients compared to subjects without renal calculus disease [9,10]. It is an observational study in which bone thickness/bone mass loss and renal calculus were correlated radiologically. Calcium metabolism is complex and involves multiple organ systems, including the gastrointestinal tract, blood plasma, intracellular and extracellular fluid, bone tissue, and kidneys. In all patients with renal calculus disease, there is derangement in renal function which, in turn, affects calcium metabolism [12]. Few other studies that measured the bone mass used lumbar vertebral bodies and femoral necks; however, as compact bones, these are affected later in the course of the disease. On the other hand, the iliac bone is a flat, cancellous bone, and changes in calcium stores of the body readily affect the bone, which would be analyzed in our study [13].

## Materials and methods

This is a non-interventional, cross-sectional, observational study conducted over a period of 12 months in the department of radiology in a tertiary care hospital. A prior institutional ethics committee approval was obtained. All patients registered at the institute who were above 18 years of age and were willing to participate in the study were included. Patients in whom renal calculus was suspected and CT of the abdomen or the kidneys, ureters, and bladder was advised by the treating physician were considered as cases. Those who were advised a CT of the abdomen for any other purpose were considered as controls. Pregnant patients, patients less than 18 years of age, and those not willing to participate were excluded from the study. Proper informed consent was taken from the patients or from their relatives if the patient was not competent to give consent (in case of altered sensorium) after explaining to them the risks and benefits of the examination. A total of 150 cases (patients with renal calculus disease) and 150 controls (patients without renal calculus) were included in the study.

All studies were performed on a Philips 64-slice Brilliance Computed Tomography unit. CT data were obtained with the field of view 350, thickness of 2 mm, pitch of 1 mm, filter standard B, window width of 60, window length of 360, matrix of 512 × 512, collimation of 1.25 mm, and reconstruction interval of 0.5 mm. During the CT scan, patients were instructed to hold their breath. The scan was performed during quiet breathing in patients who could not hold their breath. Only non-contrast-enhanced scans were performed. A weight-based, low-dose CT protocol (120 kVp, 1,000 mAs) was used. Scanning was performed from the lower thoracic level to pubic symphysis in a craniocaudal direction. After obtaining the scan with the above-mentioned parameters and protocol, documentation of the required study/CT images of the cases and controls was done. The mean iliac bone thickness (IBT) was measured on both sides at the level of the sacral ala and at the midpoint of the line joining the anterosuperior iliac spine and the sacroiliac joint. The cases and controls were divided according to their age as less than 40, 40-60, and more than 60 years. Calculi more than 5 mm in size were included whether in the kidney or the ureters. Bladder calculi were not included in the study. As there is no literature available on the Indian population on this topic, no baseline study was available for actual sample size calculation using statistical analytical formulae. Hence, the sample size was decided arbitrarily. The data were collected from the subjects who fulfilled the inclusion criteria. All data were recorded in a case record form and analyzed statistically. Spearman’s correlation test was used which is a non-parametric test that is used to measure the degree of association between two variables.

**Figure 1:**
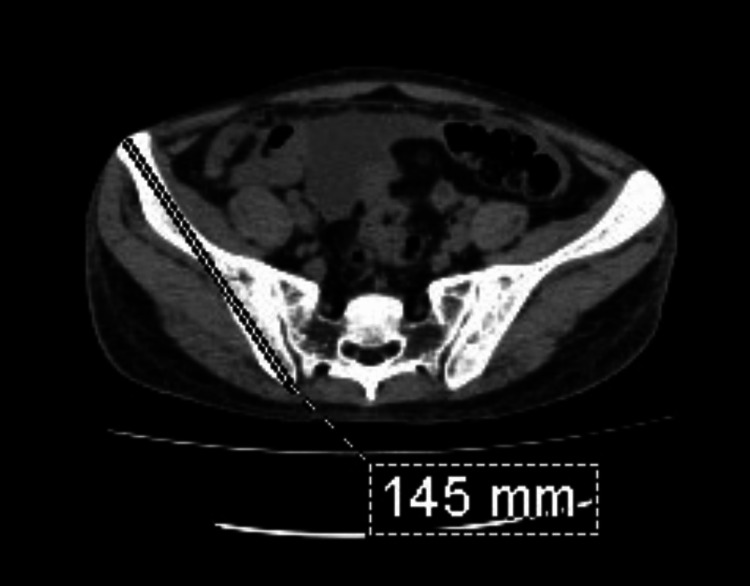
Axial CT section of the pelvis at the level of sacral ala. The line joins the anterior-superior iliac spine and the sacroiliac joint on the right side.

The following steps were used to calculate the IBT and measure the size of the calculus to define the renal calculus disease: Step one: A line joining the anterior-superior iliac spine and the sacroiliac joint was drawn (Figure 1).

**Figure 2:**
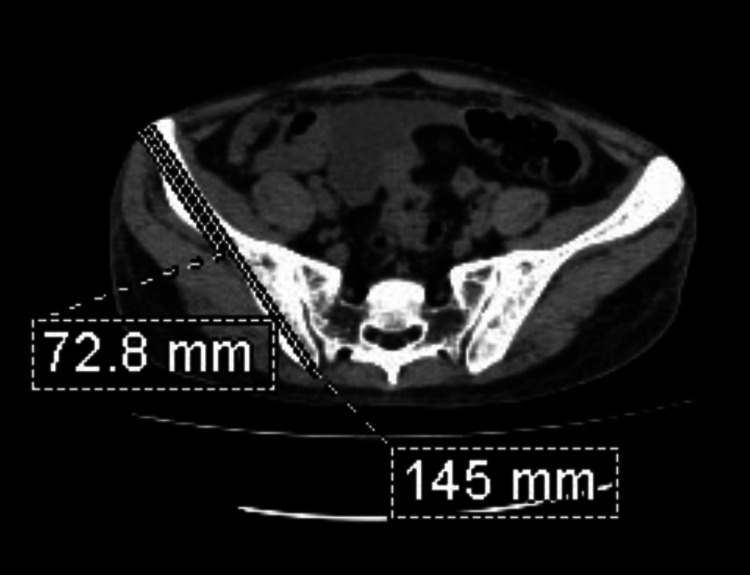
Axial CT section of the pelvis at the level of sacral ala with the midpoint of the line joining the anterior-superior iliac spine and the sacroiliac joint on the right side.

Step two: The mid-point of the line drawn was marked (Figure 2).

**Figure 3:**
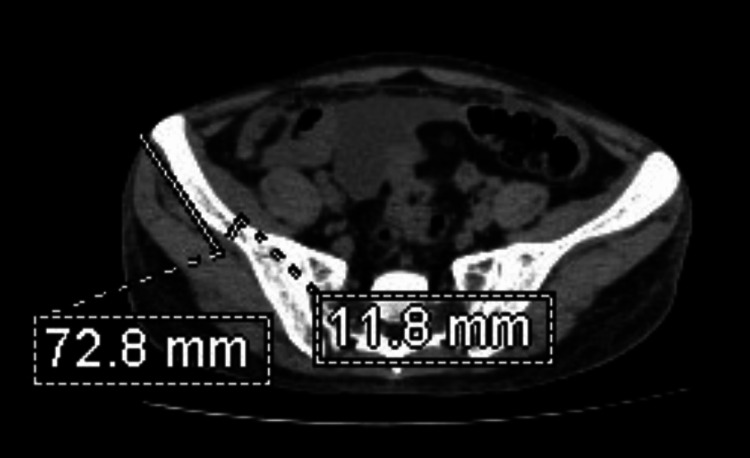
Axial CT section of the pelvis at the level of the sacral ala. Measurement of the iliac bone thickness at the midpoint of the line joining the anterior-superior iliac spine and the sacroiliac joint on the right side.

Step three: The thickness of the iliac blade at the midpoint of the line drawn was measured (Figure 3).

**Figure 4:**
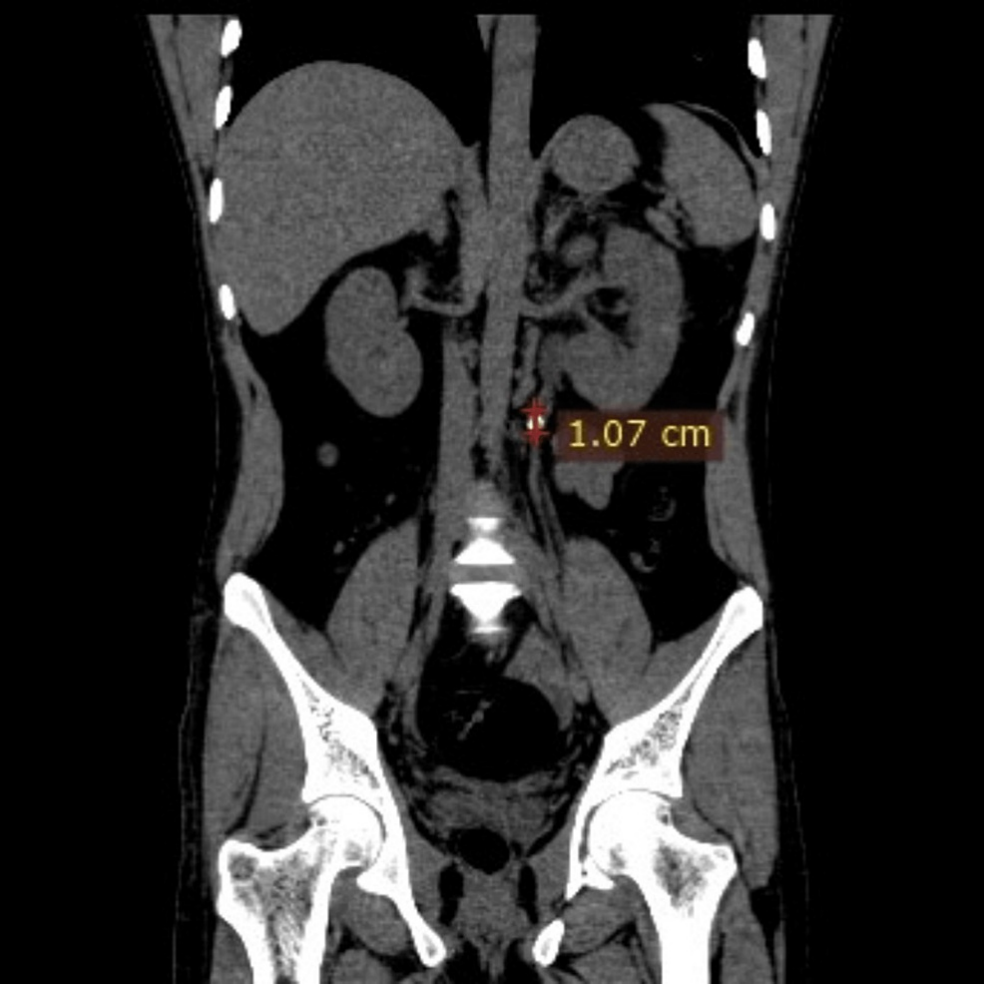
Coronal CT section of the abdomen shows a left ureteric calculus and its dimensions.

**Figure 5:**
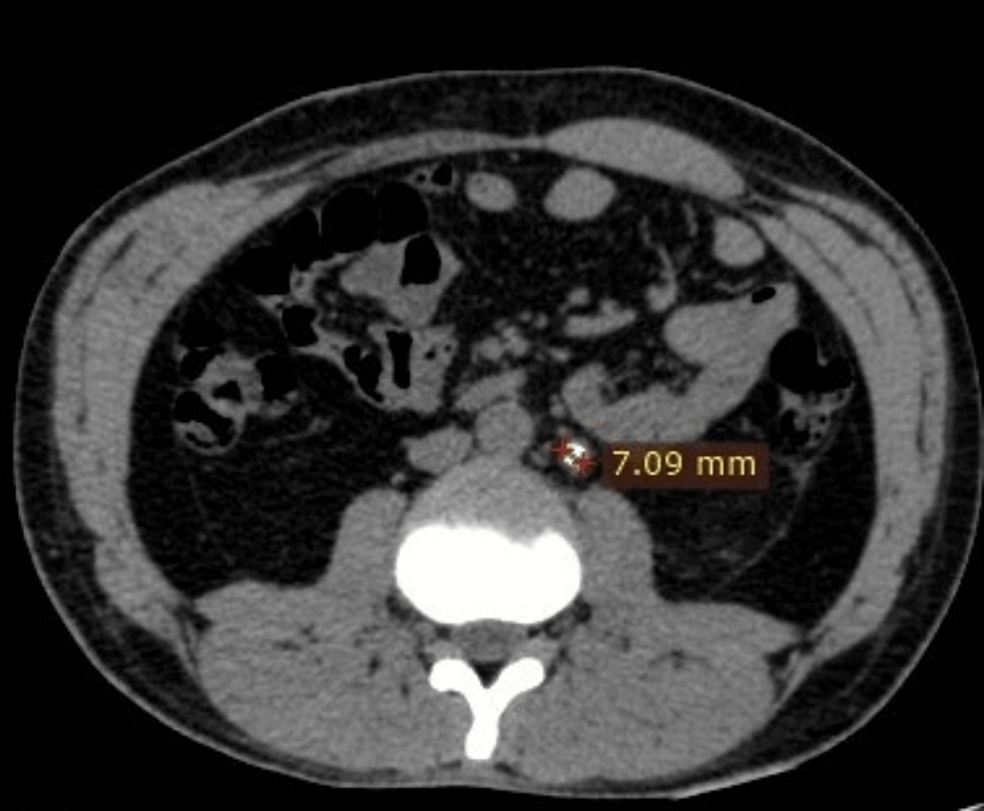
Axial CT section of the abdomen shows a left ureteric calculus and its dimensions.

Step four: The dimensions of the renal calculi were measured (Figures 4, 5).

**Figure 6:**
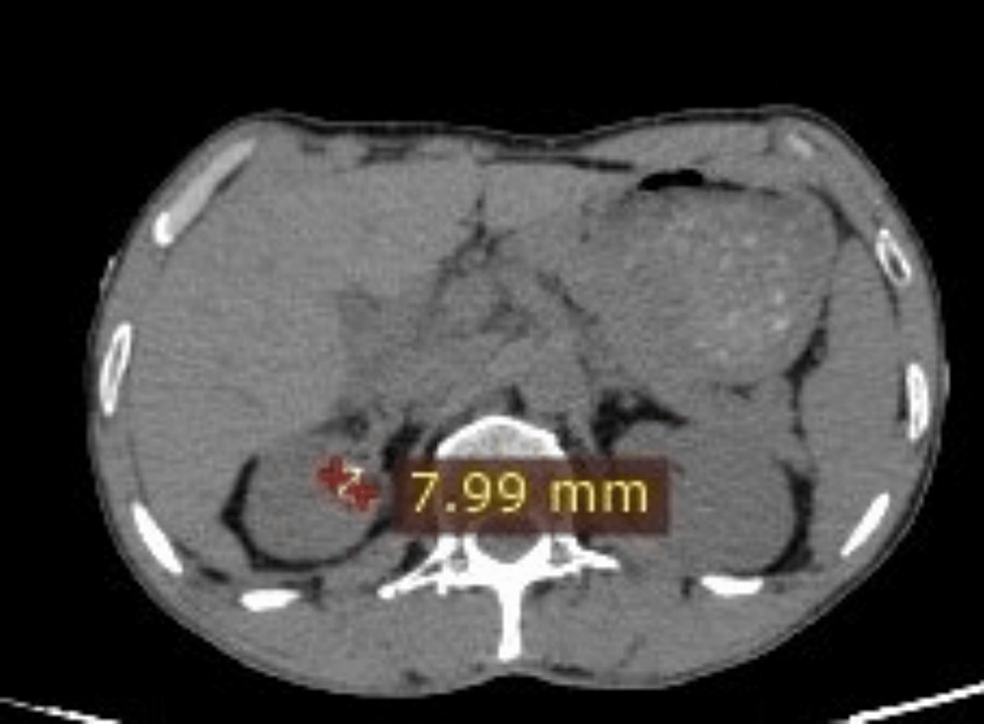
Axial CT section of the abdomen shows the measurement of a right renal calculus and its dimensions.

**Figure 7:**
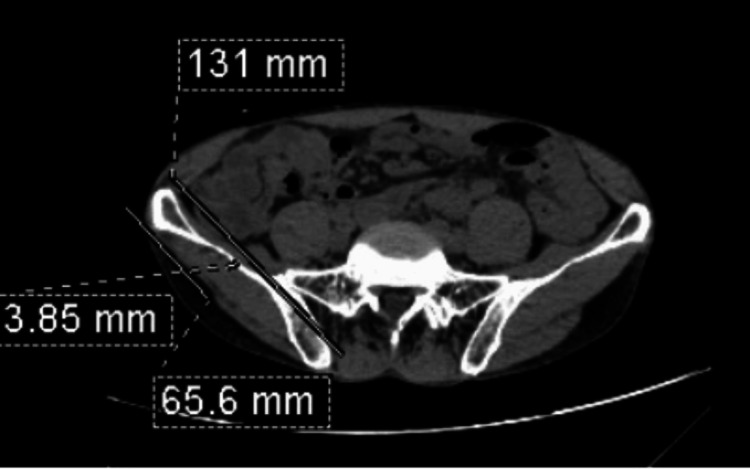
Axial CT section of the abdomen shows the measurement of the iliac bone thickness. There is cortical thinning of the iliac bone with fibrous marrow.

Another example of calculating the renal calculus size and iliac bone thickness is shown in Figure 6 and Figure 7.

After calculating the IBT on both sides, the mean value was calculated.

## Results

Out of 300 patients (150 cases and 150 controls), 84 (28%) patients were less than 40 years of age, 110 (36.67%) patients were between 40 and 60 years of age, and 106 (35.33%) were more than 60 years of age. Among 300 patients included in this study, there were equal male (150) and female (150) patients.

Out of 150 cases, 100 (66.7%) patients had renal calculi measuring more than 10 mm, and the remaining 50 (33.3%) patients had calculi measuring less than 10 mm in size. The mean IBT was significantly lower in cases (3.29 ± 0.999 mm) compared to controls (9.73 ± 1.341 mm), while there was no statistically significant difference in age in both cases and controls (Table 1). The mean IBT distribution in all age groups confirmed lower IBT in all patients with calculus disease (Table 2). In our study, changes in IBT appeared even in younger patients between 18 and 40 years of age.

**Table 1:** Mean size of the renal calculus (in mm) in cases and mean IBT (in mm) in both cases and controls. SD: standard deviation; IBT: iliac bone thickness

	Cases		Controls		P-value
	Mean	SD	Mean	SD	
Age	48.20	17.301	48.93	15.131	0.696
Size of the calculus (mm)	14.37	7.106	0	0	0
Mean IBT	3.29	0.999	9.73	1.341	0.0001

**Table 2:** Mean IBT (in mm) in cases and controls in different age groups. IBT: iliac bone thickness

	Age group <40 years		Age group 40–60 years		Age group >60 years	
Patients	Cases	Controls	Cases	Controls	Cases	Controls
Mean IBT	2.63	9.26	4.01	10.08	3.39	9.59

**Figure 8:**
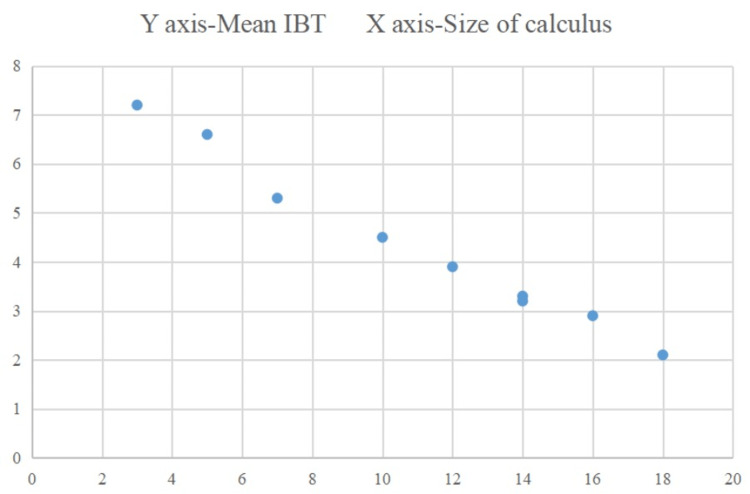
Negative correlation between the size of calculus (in mm) and the mean IBT (in mm). IBT: iliac bone thickness

In our study, there was a negative correlation between the size of renal calculus and mean IBT, which was statistically significant (Figure 8). Thus, as the size of the calculus increased, there was a significant decrease in IBT, reflecting bone mass reduction.

## Discussion

**Figure 9:**
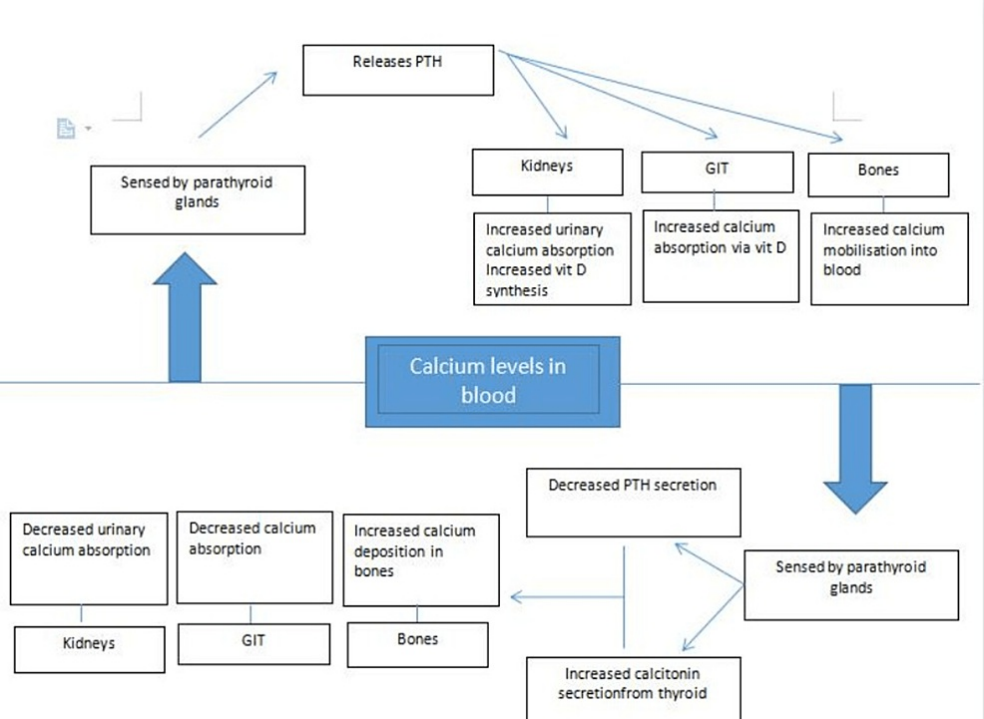
Role of multiple organ systems in calcium metabolism. PTH: parathyroid hormone; GIT: gastrointestinal

Renal calculi remain a major economic and health burden worldwide. The mechanism of stone formation is a complex process that results from several physicochemical events, including supersaturation, nucleation, growth, aggregation, and retention of urinary stone constituents within tubular cells [14]. These steps are modulated by an imbalance between factors that promote or inhibit urinary crystallization. The major composition of the renal calculus is calcium either as oxalate (70%) or phosphate (10%) [15]. Calcium metabolism is a dynamic process involving various organ systems, including the renal, musculoskeletal, endocrine, and gastrointestinal systems. Whenever the calcium levels fall in the blood pool, it is sensed by the parathyroid glands which release PTH. PTH acts on the kidneys to synthesize the active form of vitamin D which increases intestinal absorption of both calcium and phosphate. PTH also decreases urinary calcium excretion while enhancing phosphate excretion. It stimulates the osteoclasts indirectly by acting on the osteoblasts which release RANKL. Ultimately, there is mobilization of calcium into the blood. The reverse occurs when there is a rise in calcium levels where calcitonin acts by similar mechanisms to bring it back to physiological levels (Figure 9).

Although ultrasound is the first line of investigation for renal calculi due to its availability and lack of ionizing radiation, CT is considered the modality of choice for renal calculi as it offers information about the site and size of the calculi, associated complications, and other renal anomalies [16]. Causes of pain other than renal calculi can also be picked up. In our study, we compared bone mass in patients with and without renal calculi. Previously, the neck of femur and lumbar vertebrae were used for the measurement of bone mass. Both of these are compact bones that reflect the change in mass only during later stages. We propose the use of ilium for bone mass measurement as being a cancellous bone, changes in calcium stores readily affect it. Moreover, the iliac bones are already in the field of view in an abdominal CT.

In our study, the mean IBT in controls was 9.73 mm with a standard deviation of 1.341 mm, and in cases, the mean IBT was 3.29 mm with a standard deviation of 0.999 mm. This suggests that renal calculus disease is associated with renal hypercalciuria which is caused by increased bone resorption. This is reflected by decreased IBT.

The merits of the study are that it is useful to direct further management of the renal calculus disease by highlighting the importance or directing the management to the underlying deranged calcium metabolism. A reduction in bone mass can be useful in estimating osteoporosis which is a single separate risk factor in patients with renal calculi. Bone mass reduction can be a useful indicator to evaluate secondary hyperparathyroidism, osteoporosis due to hormonal deficiency, vitamin D deficiency, etc. In this study, changes in IBT were appreciated even in younger patients in the age group of 18-40 years, secondary to lifestyle changes coupled with a lack of exposure to sunlight.

The limitations of the study are, first, it does not exactly state the quantitative relation between the renal calculus size and changes in IBT. Second, it is useful only in calculi that have calcium as major composition. In patients who have urinary tract infections due to *Proteus *bacteria, which form phosphorus stones (radiolucent), bone mass reduction is not predictable by this study. Third, there was no quantitative correlation between the degree of osteoporosis and secondary hyperparathyroidism. Fourth, the aspect of diminished IBT in younger patients needs to be evaluated further.

## Conclusions

Renal calculus disease associated with hypercalciuria caused by increased bone resorption is reflected by the decreased IBT on the non-contrast CT scan. Increased bone resorption is also associated with increased risk of fractures. Our study used the iliac bone (cancellous bone) in predicting bone mass reduction which shows changes early in the course of the disease compared to the neck of the femur and lumbar vertebrae (compact bones). A decreased IBT on a single non-contrast CT scan study helps in predicting osteoporosis early and prevents the progression of the disease by early and appropriate clinical and urological intervention.
